# The Regulation of Leptin, Leptin Receptor and Pro-opiomelanocortin Expression by N-3 PUFAs in Diet-Induced Obese Mice Is Not Related to the Methylation of Their Promoters

**DOI:** 10.1186/1743-7075-8-31

**Published:** 2011-05-24

**Authors:** Chaonan Fan, Xinli Liu, Wenwen Shen, Richard J Deckelbaum, Kemin Qi

**Affiliations:** 1 Key Laboratory of Major Diseases in Children and National Key Discipline of Pediatrics (Capital Medical University), Ministry of Education, Beijing Pediatric Research Institute, Beijing Children's Hospital, Capital Medical University, Beijing, China; 2 Institute of Human Nutrition, College of Physicians and Surgeons, Columbia University, New York, USA

**Keywords:** n-3 polyunsaturated fatty acids, DNA methylation, obesity, leptin, leptin receptor, pro-opiomelanocortin, mouse

## Abstract

**Background:**

The expression of leptin is increased in obesity and inhibited by n-3 polyunsaturated fatty acids (n-3 PUFAs), but the underlying molecular mechanisms have not been firmly established.

**Methods:**

In this study, we investigated the effects of dietary n-3 PUFAs on the methylation of CpG islands in the promoter regions of the leptin, leptin-R and POMC genes, as well as the effects of n-3 PUFA status in early life on the modification of the promoters of these three genes. Male C57 BL/6J mice were fed a high-fat diet with one of four different fat types: sunflower oil (n-3 PUFA deficient), soy oil, fish oil, or a mixture of soy and fish oil (soy:fish oil = 1:1). Two low-fat diets with sunflower oil or soy oil served as controls. Female mice were fed two breeding diets, sunflower oil or a mixture of soy and fish oil (soy:fish oil = 1:1), during pregnancy and lactation to breed new pups.

**Results:**

Compared to mice fed the control diets, the expression of leptin in fat tissue and leptin-R and POMC in the hypothalamus was higher in the diet-induced obesity (DIO) mice, and the n-3 PUFAs in the diets reversed these elevated expression levels. The mean methylation levels of CpG sites in the promoter regions of the leptin and POMC genes showed no difference between the DIO and the control diet groups nor between the n-3 PUFA-containing and -deficient diet groups. For the CpG sites in the promoter regions of leptin-R, no methylation was found in any of the DIO or control groups. Feeding mice with the n-3 PUFA diet during pregnancy and lactation did not affect CpG methylation in the leptin or POMC promoters.

**Conclusions:**

Our findings indicate that promoter DNA methylation may not be related to the expression of leptin, leptin-R or its related hypothalamic satiety regulator POMC.

## Background

Obesity has become a global health epidemic. However, the molecular etiologies and mechanisms contributing to obesity have not been well characterized. Epidemiological studies have demonstrated that increased n-6 PUFAs and/or decreased n-3 PUFAs in the modern human diet have been considered to be the risk factors for the increased prevalence of chronic non-communicable diseases in modern society, including obesity, diabetes, and cardiovascular diseases [1,2]. Supplementation with fish oil n-3 PUFAs and regular exercise has been shown to reduce body fat and improve cardiovascular and metabolic health [3]. N-3 PUFAs affect both adipogenesis in vitro [4] and adipose tissue development in vivo during the gestation/lactation period. Epidemiological data from infant studies indicate that the fatty acid composition of fats is an early determinant of childhood obesity [5].

The role of n-6/n-3 PUFAs in the pathogenesis of obesity is thought to be attributable to their regulation of physiological processes, such as the synthesis and oxidation of fatty acids and the differentiation and proliferation of adipocytes, which is mediated by their ability to modulate gene transcription, messenger RNA processing and posttranslational protein modifications. However, n-3 PUFAs have been shown to have different regulatory roles from n-6 PUFAs [4,6,7]. Results from in vivo and in vitro studies have demonstrated that n-3 PUFAs are negatively associated with body leptin levels and reduce the expression of leptin [8-10]. Leptin is primarily produced by adipose tissue, and it has been shown to regulate food intake and energy expenditure through a variety of neural and endocrine mechanisms mediated by the leptin receptor (leptin-R) and its downstream satiety regulators, such as orexigenic neuropeptide Y (NPY) and anorexigenic pro-opiomelanocortin (POMC) [11]. In diet-induced obesity (DIO), the concentration of circulating leptin is elevated, and this has been interpreted as an indication of the development of leptin resistance [12], as are changes in hypothalamic leptin-R, NPY and POMC [13,14]. In rats and mice, n-3 PUFA supplementation through a high-fat diet reduces the plasma leptin concentration, leptin mRNA expression in adipose tissue, and changes the gene expression of brain satiety regulators [15,16]. Moreover, changing dietary saturated fat to n-3 PUFAs has been shown to reverse hyperleptinemia [8,9]. Therefore, n-3 PUFAs may be involved in leptin resistance in obesity, although no consistent conclusion has been reached on the relationships between them [17-19].

More recently, nutrients and other environmental factors have been shown to induce epigenetic modifications, such as CpG island methylation and histone modifications, which, in turn, determine gene silencing versus expression, leading to susceptibility to many non-communicable chronic diseases [20-24]. Some studies have demonstrated that leptin promoter methylation plays important roles in leptin expression during pre-adipocyte differentiation [25,26]. Therefore, we hypothesized that epigenetic modification of the promoters of leptin, its receptor and satiety regulators in the hypothalamus may be the mechanism that causes leptin resistance, and an imbalanced intake of n-6/n-3 PUFAs (a higher intake of n-6 PUFAs and a lower intake of n-3 PUFAs) may influence the epigenetic modification of these promoters in obesity. In this study, we utilized a high-fat DIO mouse model to investigate the effects of dietary n-3 PUFAs on CpG island methylation in the promoter regions of the leptin, leptin-R and POMC genes. Moreover, based on emerging data highlighting the possibility that the epigenetic modulation of gene expression underlies the early-life programming of an increased risk of adult-onset diseases [20,21], we determined the effects of the n-3 PUFA status on the modification of these promoters in the early life of mice and the changes in these promoters in response to later diet-induced obesity.

## Methods

### Diets

Based on the high-fat diet formula (D12492) for DIO mice from Research Diets Inc. (New Brunswick, NJ), four high-fat diets (34.9% fat by wt., 60% kcal) with different fat types were designed as DIO diets: sunflower oil (n-3 PUFA deficient), soy oil, fish oil, and a mixture of soy and fish oil (soy:fish oil = 1:1), by using the same amount of lard as the main source of fat in each diet, providing 89% of the total energy from fat. Sunflower oil was rich in n-6 PUFAs (linoleic acid, LA) (60%-70%) and deficient in n-3 PUFAs. Soy oil contained small amount of n-3 PUFAs (α-linolenic acid, ALA) (~10%) but was still rich in LA (~50%). Fish oil was a rich source of n-3 PUFAs, especially docosahexaenoic acid (DHA) and eicosapentaenoic acid (EPA) (30%). As shown in Table 1, the amount of n-3 PUFAs varied from a deficiency with the sunflower oil diet to the highest content with the fish oil diet, accounting for 0.12%, 0.79%, 1.91% and 3.07% of the total fatty acids, respectively. Meanwhile, two low-fat diets (4.3% fat by wt., 10% kcal) were also designed as controls - sunflower oil and soy oil, based on the control diet formula (D12450B) from Research Diets Inc. In order to investigate the effects of the n-3 PUFA status during both pregnancy and lactation on leptin promoter methylation in later life, we made two breeding diets were also designed: sunflower oil (n-3 PUFA deficient) and a mixture of soy and fish oil (soy:fish oil = 1:1), which were used to feed pregnant and lactating mice. All the diets were prepared by the Institute of Laboratory Animal Sciences at the Chinese Academy of Medical Sciences and stored at -20°C.

**Table 1:** Composition of the animal diets

	Control		DIO			
		
	n-3 deficient	Soy oil	n-3 deficient	Soy oil	Soy/fish oil	Fish oil
Components (g/kg)						
Lard	20	20	318	318	318	318
Sunflower oil	25	-	35	-	-	-
Soy oil	-	25	-	35	17.5	-
Fish oil	-	-	-	-	17.5	35
Fatty acid composistion (% of fat)						
Σ Saturated	27.22	28.33	41.83	42.03	43.02	44.01
Σ Momounsaturated	30.67	33.33	41.62	42.82	43.24	42.81
18:2n-6	41.14	32.39	15.51	13.36	10.65	8.59
20:4n-6	0.97	1.05	0.94	0.7	0.58	0.62
22:4n-6	-	-	-	0.3	0.6	0.9
Σ n-6 PUFAs	42.11	34.44	16.55	14.36	11.83	10.11
18:3n-3	0.09	3.89	0.12	0.79	0.17	0.11
20:5n-3	-	-	-	-	1.02	1.85
22:6n-3	-	-	-	-	0.72	1.11
Σ n-3 PUFAs	0.09	3.89	0.12	0.79	1.91	3.07
Total energy (kcal%)						
Fat	10	10	60	60	60	60
Protein	20	20	20	20	20	20
Carbohydrate	70	70	20	20	20	20

### Animals

C57 BL/6J mice (three to four weeks old, both female and male) were purchased from the Laboratory Animal Center of the Academy of Military Medical Sciences of China and housed at the animal facilities in the Center under a 12-hour (h) light/12-h dark cycle with cycles of air ventilation. After one week of recovery from transportation, the mice were fed the experimental diets. For the DIO experiment, the mice were fed one of the four DIO diets or one of the two control diets at 17:00 each day, and the body weight was measured weekly for 12 weeks. To determine the effects of the n-3 PUFA status in early life on the modification of the promoters of these genes later in life, the mice were fed one of the two types of breeding diets. After 10 weeks of feeding, the mice were mated to breed new pups, some of which were used for analysis of promoter DNA methylation at 21 days (d) after birth and the other for obesity induction by feeding n-3 PUFA-deficient DIO diet for 12 weeks. At the end of each experiment, the fasted mice were anesthetized by intraperitoneal injection of Avertin (2,2,2-tribromoethanol) (T-4840-2, Sigma-Aldrich Chemie GmbH, Steinheim, Germany) (125 mg/kg) to obtain blood samples by heart puncture. The plasma was stored at -20°C for later analysis of the leptin levels, and the white blood cells were used to determine the leptin promoter methylation. The mice were then sacrificed immediately by decapitation, and the brains and epididymal fat were dissected free of the surrounding tissue, removed, wrapped in aluminum foil and frozen in liquid N_2_. Once an entire group of animals was harvested, the tissues were transferred to -80°C until analysis. All of the animal experiments were conducted from 08:00 to 12:00 in compliance with the guidelines of the Animal Care and Use Committee of Academy of Military Medical Sciences of China.

### Analysis of plasma leptin

The plasma leptin concentration was determined by an enzyme-linked immunosorbent assay kit (Cat. No. ALX-850-317-KI01, Enzo Life Sci., Plymouth Meeting, PA, USA), according to the procedures of the manufacturer, with a detection sensitivity of 0.03 ng/L (intra-assay and inter-assay CVs of 2.9% and 2.3%, respectively).

### Quantitative RT-PCR analysis

Total RNA was extracted from mouse fat and brains with TRIzol Reagent (Cat. No. 15596-026) (Invitrogen, Carlsbad, CA, USA), and cDNA was prepared from the total RNA using the SuperScriptTM III First-Strand Synthesis System for RT-PCR (Cat. No. 18080-051) kit (Invitrogen, Carlsbad, CA, USA), according to the procedures provided. The mRNA levels of leptin, leptin-R, POMC and NPY in both fat and brains were measured with real-time quantitative RT-PCR using an ABI PRISM 7300 sequence detection system (Applied Biosystems, Foster City, CA, USA). The oligonucleotide primers and probes for leptin, leptin-R, POMC and NPY were designed and prepared by ABI Applied Biosystems (Foster City, CA, USA). The co-amplification of mouse GAPDH mRNA, an invariant internal control, was performed in all of the samples. Assays were performed in triplicate, and the results were normalized to the GAPDH mRNA levels by using the 2^ΔΔCT ^method [27].

### Bisulfite conversion and sequencing

**Figure 1:**
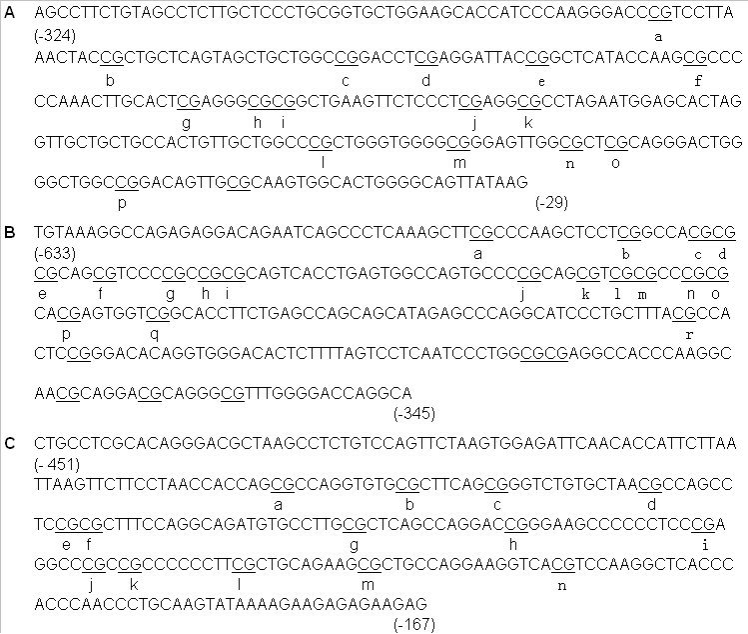
**Regions of the mouse leptin, leptin-R and POMC promoters**. The CG dinucleotides are underlined and letters (a to r) were assigned to each of the analyzed CGs. (A) The leptin promoter sequence with CpG islands spanning nucleotides -324 to -29. (B) The leptin-R promoter sequence with CpG islands spanning nucleotides -633 to -345. (C) The POMC promoter sequence with CpG islands spanning nucleotides -451 to -167.

The promoter regions of many eukaryotic genes contain stretches of CpG-rich sequences known as CpG islands. Percentage of Cs and Gs > 50%, ratio of observed to expected CG dinucleotides > 0.6 and size of CpG island sequence > 200 nucleotides were used to define CpG islands of gene promoters. As shown in Figure 1, the leptin promoter region examined covers nucleotides (nts) 29009221-29010220 and spans 16 CpGs within nts -324 to -29 (positions are given with respect to the transcription start site [TSS]). The leptin-R promoter region examined spans nts 101389512-101390511 and includes 18 CpGs within nts -633 to -345 (with respect to the TSS). The POMC promoter region examined spans nts 3954450-3955047 and includes 14 CpGs within nts -451 to -167 (with respect to the TSS). These sequence data have been submitted to the GenBank databases (http://www.ncbi.nlm.nih.gov) under accession number U18812, U46135 and NC_000078.5 respectively.

The promoter methylation of the leptin, POMC and NPY genes was analyzed by bisulfite sequencing. A DNA Purification Kit (Cat. No. DN 1008, Biofuture Group Inc, Beijing, China) was used to isolate and purify DNA from the adipose and brain tissue. Bisulfite conversion was performed using the MethylampTM DNA Modification Kit (Cat. No. P-1001, Epigentek Group Inc. Brooklyn, NY). Converted DNA was used fresh, or it was stored at -20°C. Converted DNA was amplified by nested-PCR using Taq DNA Polymerase Master Mix (Cat. No. KT201, Tiangen Biotech Inc., Beijing, China) and primer sets (Table 2) designed using Methprimer software (http://www.urogene.org/methprimer/index1.html). For leptin, the PCR conditions were 96°C for 10 minutes (min) and 45 cycles of 96°C for 1 min, 51°C for 1 min and 72°C for 1 min, followed by 10 min at 72°C. For leptin-R, the PCR conditions were 96°C for 10 min and 45 cycles of 96°C for 1 min, 55°C for 1 min and 72°C for 1 min, followed by 10 min at 72°C. For POMC, the PCR conditions were 96°C for 10 min and 45 cycles of 96°C for 1 min, 51°C for 1 min and 72°C for 1 min, followed by 10 min at 72°C. Nested PCR was performed for all three genes with the same conditions as the first step PCR. The PCR products were sequenced directly, and DNA methylation was calculated as described by Lewin et al [28].

**Table 2:** Bisulfite sequencing primers used in this study

Gene name	Forward (F) and Reverse (R) Primers	Product size (bp)
Leptin		310
	Outer F: 5'-GAGTAGTTAGGTTAGGTATGTAAAGAG-3'	
	Inner F: 5'-AGTTTTTTGTAGTTTTTTGTTTTTTG-3'	
	R: 5'-TAATAACTACCCCAATACCACTTAC-3'	
Leptin-R		294
	F: 5'-TGTAAAGGTTAGAGAGGATAGAATTAG-3'	
	Outer R: 5'-CTTATATCTTTCAAACCAACCCACCCT-3'	
	Inner R: 5'-CCAATCTACTACCTAATCCCCAAAC-3'	
POMC		258
	Outer F: 5'-TTGTAAGATTTTAGAATTAGGTTTG-3'	
	Inner F: 5'-AGTTTTTGTTTAGTTTTAAGTGGAG-3'	
	R: 5'-CTCTCTTCTTTTATACTTACAAAATTAAA-3'	

### Statistical analysis

One-way analysis of variance (ANOVA) was used to compare the means in different groups using SPSS Version 11.5 for Windows. All of the values were expressed as means ± S.D. Significant differences in leptin expression between the DIO group and the low-fat diet groups were assessed using the Student-Newman-Keuls test, and comparisons between the n-3-deficient DIO group and the n-3 PUFA-containing DIO groups were determined by Dunnett's test. For unequal variances, the Games-Howell and Dunnett's T3 tests were used to compare the differences. The Binomial test was used for comparison of DNA methylation levels.

## Results

### Changes in body weight and plasma leptin concentrations in DIO mice

After 12 weeks of feeding, the body weight in all the DIO diet groups was significantly higher than that in the control diet groups (P < 0.05). If the four DIO diet groups were compared, the body weight was lower with all n-3 PUFA-containing diets than that with the n-3 PUFA-deficient diet (P < 0.05). For food intake, differences were found among the DIO and control diet groups, showing higher energy intake with all the DIO diets than the control diets and lower energy intake with n-3 PUFA-containing DIO diets compared to n-3 PUFA-deficient DIO diet groups (P < 0.05). Plasma leptin concentrations were higher in all of the four groups of mice fed the DIO diets as compared with the mice fed the control diets (P < 0.05). Comparisons among the n-3 PUFA-deficient DIO diet and the n-3 PUFA-containing DIO diets showed that all the three n-3 PUFA-containing diets had lower levels of plasma leptin concentration. However, the difference was only shown between the fish oil DIO diet and the n-3 PUFA-deficient DIO diet (P < 0.05) (Table 3).

**Table 3:** Changes in body weight, food intake and plasma leptin concentration in DIO mice.

	Control		DIO			
		
	n-3 deficient	Soy oil	n-3 deficient	Soy oil	Soy/fish oil	Fish oil
Body weight (g)	27.69 ± 1.38	27.28 ± 1.14	34.73 ± 2.75 *	32.06 ± 2.16 * ^‡^	32.86 ± 1.96 * ^‡^	32.15 ± 1.89 * ^‡^
Leptin (μg/L)	1.39 ± 0.98	1.54 ± 1.13	8.55 ± 2.39 *	5.95 ± 3.34 *	6.79 ± 3.28 *	4.39 ± 2.09 * ^‡^
Energy intake (kcal/d/mouse) ^¶^	23.15 ± 2.59	22.99 ± 1.48	35.17 ± 3.89 *	31.37 ± 2.62 * ^‡^	32.11 ± 3.17 * ^‡^	29.54 ± 2.77 * ^‡^

3-4 week old C57BL/6J male mice were fed four DIO diets with different fat types and the control diet for 3 months. ^¶ ^Food intake was recorded at 6 weeks. Values are means ± SD, n = 15 in each group. * compared to the control diets, P < 0.05; ^‡ ^compared to the n-3 deficient DIO diet, P < 0.05.

### Effects of n-3 PUFAs on the gene expression of leptin, leptin-R, POMC and NPY in DIO mice

**Figure 2:**
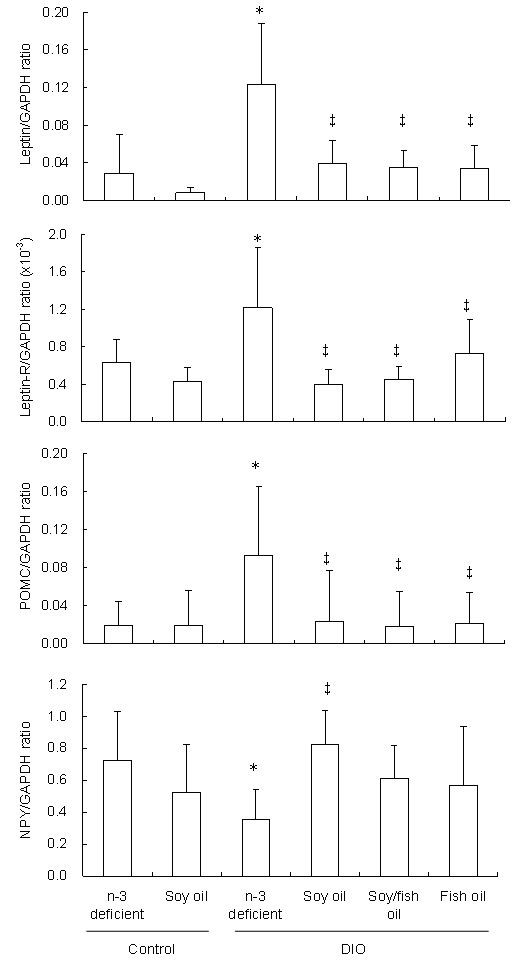
**The effects of n-3 PUFAs on the mRNA expression of leptin, leptin-R, POMC and NPY in DIO mice**. Male C57BL/6J mice, 3-4 weeks old, were fed one of four DIO diets with different fat types or a control diet for 3 months. Real-time quantitative RT-PCR was used to measure the mRNA levels of leptin in epididymal fat and those of leptin-R, POMC and NPY in the hypothalamus. The data were normalized to GAPDH mRNA levels using the 2^ΔΔCT ^method. Values are means ± SD, n = 15 in each group. * compared to the control diets, P < 0.05; ^‡ ^compared to the n-3 deficient DIO diet, P < 0.05.

As shown in Figure 2, compared to the n-3-deficient and soy oil diet groups, the mRNA expression levels of leptin, leptin-R and POMC were higher (P < 0.05), and that of NPY was lower, only in the n-3-deficient DIO diet group (P < 0.05); no changes occurred in the other three n-3 PUFA-containing DIO diet groups. These results indicate that the addition of n-3 PUFAs to diets reverses both the increased expression of leptin, leptin-R and POMC and the decreased expression of NPY.

### Effects of n-3 PUFAs on the methylation of CpG islands in the promoters of the leptin, leptin-R and POMC genes in DIO mice

The mean methylation level of the 16 and 14 CpG sites in the promoter regions of the leptin and POMC genes ranged from 72% to 77% and 69% to 71%, respectively, and showed no significant differences between the DIO and the control mice (Table 4 and Table 5). However, an increased methylation level was indicated in many CpG sites of the leptin promoter in DIO mice compared to the control mice. Furthermore, the n-3 PUFA-containing diets seemed to reduce the methylation level of leptin promoter in DIO mice. For the 18 CpG sites in the promoter region of leptin-R, no methylation was found in any of the DIO or control groups.

**Table 4:** Quantitative methylation analysis of the promoter region of the leptin gene.

Diets	CpG site (position)																Total
		
	a	b	c	d	e	f	g	h	i	j	k	l	m	n	o	p	
DIO																	
n-3 deficient	66	80	63	71	80	68	78	83	67	80	77	81	82	78	89	93	77
Soy oil	65	78	63	69	79	68	80	83	66	78	79	81	82	81	90	95	77
Soy/Fish oil	62	78	62	68	77	66	78	82	65	76	76	79	81	79	89	96	76
Fish oil	63	75	61	66	75	66	77	82	64	76	76	79	80	78	88	93	75
Control																	
n-3 deficient	58	69	57	65	74	61	75	80	67	71	70	74	78	74	88	93	72
Soy oil	60	73	56	64	72	63	74	78	63	73	73	77	80	78	87	94	73

3-4 weeks old C57BL/6J male mice were fed four DIO diets with different fat types and the control diet for 3 months. Genomic DNA isolated from epididymal fat was analyzed for methylation status of 16 CpG sites at the indicated positions of the leptin gene promoter spanning nucleotides from -324 to -29 relative to transcription start site. Data for each CpG site represent the mean percentage level (0% = nonmethylated, 100% = methylated) determined from 15 mice in each group.

**Table 5:** Quantitative methylation analysis of the promoter region of the POMC gene.

Diets	CpG site (position)														Total
		
	a	b	c	d	e	f	g	h	i	j	k	l	m	n	
DIO															
n-3 deficient	81	80	61	71	75	81	80	47	50	75	63	63	71	73	69
Soy oil	80	80	61	73	78	83	81	48	48	77	67	65	75	76	71
Soy/Fish oil	81	81	62	73	78	82	81	48	49	75	64	62	72	73	70
Fish oil	78	77	57	71	79	83	82	45	52	76	56	65	73	75	69
Control															
n-3 deficient	80	80	61	72	78	81	80	47	50	76	65	63	73	73	70
Soy oil	78	78	58	70	78	81	81	46	49	76	64	65	74	75	70

3-4 week old C57BL/6J male mice were fed four DIO diets with different fat types and the control diet for 3 months. Genomic DNA isolated from epididymal fat tissue was analyzed for methylation status of 14 CpG sites at the indicated positions of the POMC gene promoter spanning nucleotides -660 to -53 relative to transcription start site. Data for each CpG site represent the mean percentage level (0% = nonmethylated, 100% = methylated) determined from 15 mice in each group.

### Effects of n-3 PUFAs on the methylation of CpG islands in the promoters of the leptin, leptin-R and POMC genes in early life and their changes in later diet-induced obesity

For 21-day-old mice fed the n-3 PUFA-containing diet during maternal pregnancy and lactation, the mean methylation levels of 16 and 14 CpG sites in the promoter regions of the leptin and POMC genes (88% and 70%), respectively, did not change, as compared to those in the n-3 PUFA-deficient diet group (86% and 69%). Moreover, after three months of obesity induction, the mean methylation levels of the CpG sites in the promoter regions of the leptin and POMC genes did not show any differences between the n-3 PUFA-deficient diet (83% and 65.2%) and n-3 PUFA-containing diet groups (81% and 65.1%). For the 18 CpG sites in the promoter region of the leptin-R gene, no methylation was found in any of the DIO or control groups.

## Discussion

In the present study we found that the expression of leptin in DIO mice was higher than in low-fat fed mice, and the addition of n-3 PUFAs to the diet reduced the expression of leptin. Moreover, we found that DIO mice had higher mRNA expression levels of leptin-R and POMC and lower expression levels of NPY, and the expression of all three of the genes was normalized by adding n-3 PUFAs to the diet. It has been found that n-3 PUFA supplementation in high-fat diets fed to rats and mice reduces the plasma leptin concentration and leptin mRNA expression in adipose tissue, and changing the dietary saturated fat to n-3 PUFAs reverses hyperleptinemia [8,9,29], which agrees with our results. In contrast, increased leptin expression in response to n-3 PUFAs has also been reported in rodents and in primary cultured rat adipocytes [17,18]. Still, no consistent conclusion has been reached of the effects of n-3 PUFAs on the expression of leptin-R and its related neuropeptides in obesity. The hypothalamus leptin-R expression in DIO rodents is increased with either shorter feeding periods (6 to 7 weeks) or longer feeding periods (19 weeks) [9,15], whereas the expressions of POMC and NPY vary with increase, reduction or no change independent of the feeding duration [9,15,16,30]. All these discrepancies need to be clarified, although they are likely due to the differences in diet composition, dietary status (fed or fasted), the period of obesity induction as well as fluctuations with diurnal rhythm [9,15,16,30].

The homeostatic response of POMC and NPY mRNA expressions to the high leptin levels in the DIO diet groups should then, at least as one important factor, have been associated with satiety and have worked to normalize weight (and fat) gain. In this study, food intake was increased in DIO diet groups although POMC and NPY mRNA expressions were increased and reduced respectively with DIO diet feeding, compared with the control diets. This suggests that the response of POMC and NPY expressions in DIO mice is insufficient to maintain energy homeostasis. If this response lasts for a longer period, leptin resistance may occur. As far as the effects of fat type concerned, in this study, introduction of n-3 PUFAs to DIO diets reversed the abnormal expressions of the above genes, accompanying reduced energy intake and body weight. This is in keeping with many other reports demonstrating that supplementing the diet with n-3 PUFAs can attenuate weight gain and reduce body fat by altering gene expression in a range of tissues that favour increased fat oxidation and reduced fat deposition, and reductions in food intake and apoptosis of adipocytes [31].

The mechanisms underlying the abnormal expression of these genes and the regulatory roles of n-3 PUFAs are complicated and multiple, and they are still far from being fully understood. Several lines of evidence have suggested that the expression of genes controlling energy homeostasis could be regulated by epigenetic mechanisms, which may play a role in the development of obesity [32,33]. Recent studies have demonstrated that leptin promoter methylation plays important roles in leptin expression. In vivo studies in humans and mice have indicated that leptin promoter methylation is normally imposed during postzygotic development, and that this epigenetic mark may function in modulating the leptin expression [26]. Melzner et al. reported that CpGs in the proximal leptin promoter were highly methylated in pre-adipocytes, and during maturation toward terminally differentiated adipocytes, this promoter region was found to be highly demethylated [25]. This suggests that the methylation of CpGs inhibits leptin expression, whereas their demethylation activates leptin expression.

Very recently, Milagro et al. reported that high-fat diet-induced obesity modifies the methylation pattern of the leptin promoter in rats, although only one CpG site was slightly methylated [34]. However, Okada et al. have demonstrated that the diet-induced up-regulation of leptin expression was not mediated by changes of the methylation of its promoter [35]. In the present study, a declining trend was shown in the level of leptin promoter methylation in DIO mice as compared with low-fat control mice and the addition of n-3 PUFAs to the diets seemed to reduce the level of leptin promoter methylation, although the significant difference was not reached statistically. Whether this is attributable for shorter feeding periods (12 weeks) in our study needs to be clarified by inducing DIO mice for longer feeding periods in the future. However, no changes were found for the methylation of the leptin-R and POMC promoters in DIO mice, and dietary n-3 PUFA supplementation did not affect the promoter methylation of these genes. In addition, we also tested the leptin promoter methylation in peripheral blood cells in obese preschool children and found no changes, as compared to lean children (unpublished data). Taken together, our findings indicate that the transcriptional regulation of leptin, leptin-R and POMC by n-3 PUFAs in DIO mice is not associated with the methylation of their promoters.

Extensive human epidemiological data have indicated that nutrition during critical periods of development can influence adult susceptibility to non-communicable chronic diseases, and the underlying mechanisms are defined as epigenetic modifications that determine gene silencing versus expression [20-24]. Changes in leptin expression during perinatal life are associated with adult leptin resistance and adipogenic and diabetogenic phenotypes. Moreover, leptin treatment in adult female rat offspring that were born following maternal undernutrition failed to reduce the food intake, and weight loss was diminished compared with leptin-treated offspring of dams fed ad libitum throughout pregnancy. These results suggest that prenatal nutrition can shape future susceptibility to obesity through alterations in leptin sensitivity and changes in energy metabolism during adult life [36,37]. Moreover, the manipulation of the postnatal diet has been shown to limit the adverse outcomes of fetal programming, as a postnatal diet enriched with n-3 fatty acids prevented pre-programmed hyperleptinemia and hypertension [5,38]. To investigate whether the status of n-3 PUFAs in early life affected the methylation status of the leptin, leptin-R or POMC promoters, we fed mice with the diets rich in n-3 PUFAs during pregnancy and lactation, and no changes were observed in the promoter methylation levels of these three genes in the offspring at the age of infancy and adulthood.

Recently, much attention has been focused on histone modifications because of their critical role in regulating gene expression and their active involvement in a number of cellular processes, such as mitosis and cellular differentiation [39]. Histone H3 and H4 hyperacetylation in promoter regions is associated with gene activation in organisms ranging from yeast to mammals, and transcriptionally active euchromatin regions are highly enriched in acetylated histones, whereas histone methylation can have both positive and negative effects on transcription, depending on the site of methylation [40-42]. Recent studies have revealed that changes in histone modifications are a key component of an epigenetic network controlling adipogenesis and energy homeostasis. Studies of the role of epigenetic histone modifications in human nutrition and obesity will be a fruitful area for further research [32,43,44].

## Conclusion

To summarize, our findings indicate that promoter DNA methylation does not contribute directly to the changes in the expression of leptin, leptin-R or its related hypothalamic POMC. More detailed studies of the epigenetic changes associated with leptin resistance in obesity are warranted to gain insight into the link between n-3 PUFAs and obesity. Histone modifications in the promoters of leptin, leptin-R and its related satiety regulators will be important to elucidate the regulatory influence of n-3 PUFAs on leptin expression in obesity.

## List of abbreviations

DIO: diet-induced obesity; leptin-R: leptin receptor; NPY: neuropeptide Y; POMC: pro-opiomelanocortin; PUFAs: polyunsaturated fatty acids;

## Competing interests

The authors declare that they have no competing interests.

## Authors' contributions

CF carried out DNA methylation studies, participated in the study design and drafted the manuscript. XL carried out the studies of n-3 PUFAs and DNA methylation in DIO mice, and participated in the statistical analysis. WS carried out studies of n-3 PUFAs in early life and DNA methylation, and participated in the statistical analysis. RJD participated in the study design and helped to review the manuscript. KQ conceived of the study, and participated in its design and coordination and helped to draft the manuscript. All authors read and approved the final manuscript.

## References

[B1] Ailhaud G Massiera F Weill P Legrand P Alessandri JM Guesnet P Temporal changes in dietary fats: role of n-6 polyunsaturated fatty acids in excessive adipose tissue development and relationship to obesity Prog Lipid Res 2006 45 203 236 10.1016/j.plipres.2006.01.003 16516300

[B2] Simopoulos AP Evolutionary aspects of diet, the omega-6/omega-3 ratio and genetic variation: nutritional implications for chronic diseases Biomed Pharmacother 2006 60 502 507 10.1016/j.biopha.2006.07.080 17045449

[B3] Hill AM Buckley JD Murphy KJ Howe PR Combining fish-oil supplements with regular aerobic exercise improves body composition and cardiovascular disease risk factors Am J Clin Nutr 2007 85 1267 1274 17490962 10.1093/ajcn/85.5.1267

[B4] Madsen L Petersen RK Kristiansen K Regulation of adipocyte differentiation and function by polyunsaturated fatty acids Biochim Biophys Acta 2005 1740 266 286 15949694 10.1016/j.bbadis.2005.03.001

[B5] Ailhaud G Guesnet P Fatty acid composition of fats is an early determinant of childhood obesity: a short review and an opinion Obes Rev 2004 5 21 26 10.1111/j.1467-789X.2004.00121.x 14969504

[B6] Davidson MH Mechanisms for the hypotriglyceridemic effect of marine omega-3 fatty acids Am J Cardiol 2006 98 27 33 10.1016/j.amjcard.2005.12.024 16919514

[B7] Deckelbaum RJ Worgall TS Seo T n-3 fatty acids and gene expression Am J Clin Nutr 2006 83 6 Suppl 1520S 1525S 16841862 10.1093/ajcn/83.6.1520S

[B8] Ukropec J Reseland JE Gasperikova D Demcakova E Madsen L Berge RK Rustan AC Klimes I Drevon CA Sebökova E The hypotriglyceridemic effect of dietary n-3 FA is associated with increased beta-oxidation and reduced leptin expression Lipids 2003 38 1023 1029 10.1007/s11745-006-1156-z 14669966

[B9] Wang H Storlien LH Huang XF Effects of dietary fat types on body fatness, leptin, and ARC leptin receptor, NPY, and AgRP mRNA expression Am J Physiol Endocrinol Metab 2002 282 E1352 1359 12006366 10.1152/ajpendo.00230.2001

[B10] Winnicki M Somers VK Accurso V Phillips BG Puato M Palatini P Pauletto P Fish-rich diet, leptin, and body mass Circulation 2002 106 289 291 10.1161/01.CIR.0000025241.01418.4D 12119240

[B11] Bouret SG Early life origins of obesity: role of hypothalamic programming J Pediatr Gastroenterol Nutr 2009 48 Suppl 1 S31 38 10.1097/MPG.0b013e3181977375 19214056

[B12] Considine RV Human leptin: an adipocyte hormone with weight-regulatory and endocrine functions Semin Vasc Med 2005 5 15 24 10.1055/s-2005-871738 15968576

[B13] Bjørbaek C Central leptin receptor action and resistance in obesity J Investig Med 2009 57 789 794 20029269 10.231/JIM.0b013e3181bb0d49 PMC2798145

[B14] Levin BE Dunn MA Dysregulation of arcuate nucleus preproneuropeptide Y mRNA in diet-induced obese rats Am J Physiol 1997 272 1365 1370 10.1152/ajpregu.1997.272.5.R1365 9176325

[B15] Huang XF Xin X McLennan P Storlien L Role of fat amount and type in ameliorating diet-induced obesity: insights at the level of hypothalamic arcuate nucleus leptin receptor, neuropeptide Y and pro-opiomelanocortin mRNA expression Diabetes Obes Metab 2004 6 35 44 10.1111/j.1463-1326.2004.00312.x 14686961

[B16] Dziedzic B Szemraj J Bartkowiak J Walczewska A Various dietary fats differentially change the gene expression of neuropeptides involved in body weight regulation in rats J Neuroendocrinol 2007 19 364 373 10.1111/j.1365-2826.2007.01541.x 17425611

[B17] Peyron-Caso E Taverna M Guerre-Millo M Véronèse A Pacher N Slama G Rizkalla SW Dietary (n-3) polyunsaturated fatty acids up-regulate plasma leptin in insulin-resistant rats J Nutr 2002 132 2235 2240 12163668 10.1093/jn/132.8.2235

[B18] Pérez-Matute P Marti A Martínez JA Fernández-Otero MP Stanhope KL Havel PJ Moreno-Aliaga MJ Eicosapentaenoic fatty acid increases leptin secretion from primary cultured rat adipocytes: role of glucose metabolism Am J Physiol Regul Integr Comp Physiol 2005 288 R1682 1688 10.1152/ajpregu.00727.2004 15650121

[B19] Kratz M von Eckardstein A Fobker M Buyken A Posny N Schulte H Assmann G Wahrburg U The impact of dietary fat composition on serum leptin concentrations in healthy nonobese men and women J Clin Endocrinol Metab 2002 87 5008 5014 10.1210/jc.2002-020496 12414865

[B20] Dolinoy DC Weidman JR Jirtle RL Epigenetic gene regulation: linking early developmental environment to adult disease Reprod Toxicol 2007 23 297 307 10.1016/j.reprotox.2006.08.012 17046196

[B21] Burdge GC Hanson MA Slater-Jefferies JL Lillycrop KA Epigenetic regulation of transcription: a mechanism for inducing variations in phenotype (fetal programming) by differences in nutrition during early life? Br J Nutr 2007 97 1036 1046 10.1017/S0007114507682920 17381976 PMC2211525

[B22] Zeisel SH Nutrigenomics and metabolomics will change clinical nutrition and public health practice: insights from studies on dietary requirements for choline Am J Clin Nutr 2007 86 542 548 17823415 10.1093/ajcn/86.3.542 PMC2430757

[B23] Cooney CA Dave AA Wolff GL Maternal methyl supplements in mice affect epigenetic variation and DNA methylation of offspring J Nutr 2002 132 Suppl 2393S 400S 12163699 10.1093/jn/132.8.2393S

[B24] Waterland RA Is epigenetics an important link between early life events and adult disease? Horm Res 2009 71 Suppl 1 13 16 19153498 10.1159/000178030

[B25] Melzner I Scott V Dorsch K Fischer P Wabitsch M Brüderlein S Hasel C Möller P Leptin gene expression in human preadipocytes is switched on by maturation-induced demethylation of distinct CpGs in its proximal promoter J Biol Chem 2002 277 45420 45427 10.1074/jbc.M208511200 12213831

[B26] Stoger R In vivo methylation patterns of the leptin promoter in human and mouse Epigenetics 2006 1 155 162 10.4161/epi.1.4.3400 17965621

[B27] Livak KJ Schmittgen TD Analysis of relative gene expression data using real-time quantitative PCR and the 2(-Delta Delta C(T)) Method Methods 2001 25 402 408 10.1006/meth.2001.1262 11846609

[B28] Lewin J Schmitt AO Adorján P Hildmann T Piepenbrock C Quantitative DNA methylation analysis based on four-dye trace data from direct sequencing of PCR amplificates Bioinformatics 2004 20 3005 3012 10.1093/bioinformatics/bth346 15247106

[B29] Selenscig D Rossi A Chicco A Lombardo YB Increased leptin storage with altered leptin secretion from adipocytes of rats with sucrose-induced dyslipidemia and insulin resistance: effect of dietary fish oil Metabolism 2010 59 787 795 10.1016/j.metabol.2009.09.025 20005540

[B30] Gout J Sarafian D Tirard J Blondet A Vigier M Rajas F Mithieux G Begeot M Naville D Leptin infusion and obesity in mouse cause alterations in the hypothalamic melanocortin system Obesity (Silver Spring) 2008 16 1763 1769 10.1038/oby.2008.303 18551122

[B31] Buckley JD Howe PRC Anti-obesity effects of omega-3 polyunsaturated fatty acids Obesity Review 2009 10 648 659 10.1111/j.1467-789X.2009.00584.x 19460115

[B32] Campión J Milagro FI Martínez JA Individuality and epigenetics in obesity Obes Rev 2009 10 383 392 10.1111/j.1467-789X.2009.00595.x 19413700

[B33] Lomba A Milagro FI García-Díaz DF Marti A Campión J Martínez JA Obesity induced by a pair-fed high fat sucrose diet: methylation and expression pattern of genes related to energy homeostasis Lipids Health Dis 2010 9 60 10.1186/1476-511X-9-60 20534152 PMC2909242

[B34] Milagro FI Campión J García-Díaz DF Goyenechea E Paternain L Martínez JA High fat diet-induced obesity modifies the methylation pattern of leptin promoter in rats J Physiol Biochem 2009 65 1 9 10.1007/BF03165964 19588726

[B35] Okada Y Sakaue H Nagare T Kasuga M Diet-induced up-regulation of gene expression in adipocytes without changes in DNA methylation Kobe J Med Sci 2009 54 E241 249 19628964

[B36] Plagemann A Harder T Hormonal programming in perinatal life: leptin and beyond Br J Nutr 2009 101 151 152 10.1017/S0007114508024021 18680628

[B37] Krechowec SO Vickers M Gertler A Breier BH Prenatal influences on leptin sensitivity and susceptibility to diet-induced obesity J Endocrinol 2006 189 355 363 10.1677/joe.1.06679 16648302

[B38] Wyrwoll CS Mark PJ Mori TA Puddey IB Waddell BJ Prevention of programmed hyperleptinemia and hypertension by postnatal dietary omega-3 fatty acids Endocrinology 2006 147 599 606 16210371 10.1210/en.2005-0748

[B39] Wolffe AP Transcriptional regulation in the context of chromatin structure Essays Biochem 2001 37 45 57 11758456 10.1042/bse0370045

[B40] Pokholok DK Harbison CT Levine S Cole M Hannett NM Lee TI Bell GW Walker K Rolfe PA Herbolsheimer E Zeitlinger J Lewitter F Gifford DK Young RA Genome-wide map of nucleosome acetylation and methylation in yeast Cell 2005 122 517 527 10.1016/j.cell.2005.06.026 16122420

[B41] Schübeler D MacAlpine DM Scalzo D Wirbelauer C Kooperberg C van Leeuwen F Gottschling DE O'Neill LP Turner BM Delrow J Bell SP Groudine M The histone modification pattern of active genes revealed through genome-wide chromatin analysis of a higher eukaryote Genes Dev 2004 18 1263 1271 10.1101/gad.1198204 15175259 PMC420352

[B42] Myers FA Evans DR Clayton AL Thorne AW Crane-Robinson C Targeted and extended acetylation of histones H4 and H3 at active and inactive genes in chicken embryo erythrocytes J Biol Chem 2001 276 20197 20205 10.1074/jbc.M009472200 11274167

[B43] Okamura M Inagaki T Tanaka T Sakai J Role of histone methylation and demethylation in adipogenesis and obesity Organogenesis 2010 6 24 32 10.4161/org.6.1.11121 20592862 PMC2861740

[B44] Musri MM Corominola H Casamitjana R Gomis R Párrizas M Histone H3 lysine 4 dimethylation signals the transcriptional competence of the adiponectin promoter in preadipocytes J Biol Chem 2006 281 17180 17188 10.1074/jbc.M601295200 16613853

